# Mother adversity and co-residence time impact mother–child similarity in genome-wide and gene-specific methylation profiles

**DOI:** 10.1186/s13148-024-01655-5

**Published:** 2024-03-20

**Authors:** Lucía Labaut, Agustín Lage-Castellanos, María José Rodrigo, Silvia Herrero-Roldán, Colter Mitchell, Jonah Fisher, Inmaculada León

**Affiliations:** 1 Instituto Universitario de Neurociencia, Campus de Guajara, Universidad de La Laguna, 38201 La Laguna, Tenerife Spain; 2 Facultad de Psicología, Universidad de La Laguna, San Cristóbal de La Laguna, Spain; 3 Facultad de Ciencias Sociales Aplicadas y de la Comunicación, UNIE Universidad, Madrid, Spain; 4 Institute for Social Research, University of Michigan, Ann Abor, MI USA; 5 Department of NeuroInformatics, Cuban Center for Neuroscience, Havana, Cuba; 6 Department of Cognitive Neuroscience, Faculty of Psychology and Neuroscience, Maastricht University, Maastricht, The Netherlands

**Keywords:** Epigenetics, Mother–child covariance, Maternal adversity, Living together, EWAS, Stress-related genes

## Abstract

**Background:**

The effects of adverse life events on physical and psychological health, with DNA methylation (DNAm) as a critical underlying mechanism, have been extensively studied. However, the epigenetic resemblance between mother and child in the context of neglectful caregiving, and whether it may be shaped by the emotional impact of maternal stressful events and the duration of co-residence (indexed by child age), remains unknown. The present study examined mother–child similarity in methylation profiles, considering the potential effect of mother adversity, mother empathy, neglect-control group, child age (an index of years of mother–child co-residence), and mother age. Using Illumina Epic arrays, we quantified DNAm in 115 mother–child saliva samples. We obtained a methylation similarity index by computing correlation coefficients between methylation profiles within dyads, for the entire epigenome, and five specific genes related to stress and empathy: NR3C1, FKPB5, OXTR, SCL6A4, and BDNF.

**Results:**

The methylation profiles of the mother–child familial pairs significantly correlated as compared to mother–child random pairs for the entire epigenome and NR3C1, FKBP5, OXTR and BDNF genes. Next, multiple linear regression models observed associations of mother adversity, child age, and neglect-control group on mother–child methylation similarity, only significant in mother–child familial pairs, after correcting for multiple comparisons. Higher mother adversity was associated with lower mother–child methylation similarity for the epigenome-wide analysis, for the BDNF gene, and in the neglect-control group for the OXTR gene. In turn, being an older child (longer co-residence) was associated with higher mother–child methylation similarity.

**Conclusions:**

Mother adversity and co-residence time are modulating factors in the intergenerational methylation process that offer a window into development-dependent adaptations that can be affected by both hereditary and environmental factors, significantly observed only in biological dyads. A twofold implication for child well-being emerges, one is positive in that children of mothers exposed to life adversity or neglect did not necessarily inherit their methylation patterns. The other is concerning due to the influence of time spent living together, which affects similarity with the mother and potentially increases the risk of inheriting an epigenetic profile associated with future dysfunctional parenting patterns. This underscores the importance of the 'the earlier, the better' recommendation by the Child Protection System, which is not always followed.

## Background

Growing evidence underscores the enduring and profound impact of life adversity on children and adults' mental and physical well-being [1]. DNA methylation (DNAm), a pivotal epigenetic process, has been posited as a molecular mechanism underpinning this association [2, 3]. Nonetheless, the precise biological pathways through which these mechanisms operate in the context of maladaptive parenting, involving poor affective interactions between mothers and children, remain a relatively unexplored area. Building upon this biological perspective, the present study delves into the shared methylation patterns in mother–child dyads, employing comprehensive genome-wide and gene-specific analyses in extreme mothering, such as neglectful caregiving. Additionally, this study seeks to unravel how maternal exposure to adversity, trait empathy, and child age as an index of years of mother–child co-residence influence the similarity methylation patterns between mothers and their offspring. In doing so, this investigation has the potential to shed light on the circumstances that influence the transmission from one generation to the next.

Neglect is the most common and severe form of child maltreatment, which consists of the caregivers’ failure to provide the child with food, clothing, shelter, medical care, supervision, or emotional support [4, 5]. Being a mother with neglectful care is typically linked to having suffered childhood maltreatment [6] and exposure to adverse events [7] in her own life. Often these experiences are followed by negative effects on mental and physical health throughout life [8, 9] and emotional difficulties such as a lack of empathy and alexithymia [10]. Being a neglected child involves, in turn, experiencing stressful care [11], not being treated empathically, and carrying a cumulative risk of behavioral and mental problems [12].

The sparse epigenetic evidence in neglectful caregiving with genome-wide analysis has shown that mothers and their children shared nine differentially methylated regions and some stress-related genes, compared to control supportive caregiving [13]. In turn, higher levels of empathy in a population of mothers reduced the epigenetic aging acceleration, especially in mothers showing neglectful caregiving [14]. In non-negligence contexts, the influence of mother stress on methylation covariance between mother and offspring pairs has been analyzed, particularly for specific genes, with heterogeneous results depending on the genes and the type of population. In parents exposed to the Holocaust and their adult offspring, inversed methylation variations were found on the same sites for the gene FKBP5 [15], suggesting interrelations far from a replica, between the mother and child epigenetic changes. For NR3C1, a gene associated with stress response, the methylation levels of mothers and children in the Exon 1F Promoter region were positively correlated only in the presence of mothers’ interpersonal violence-related post-traumatic stress disorder [16]. Positive correlations between mothers and adolescents were identified involving the NR3C1 gene and the serotonin transporter 5HTT gene [17, 18].

Studies relating variations in maternal early caring with DNAm changes have pointed out the critical role of the oxytocin receptor gene (OXTR) in mother–infant bonding [19, 20]. In young infants, methylation of the OXTR was associated with maternal structuring behaviors, and more child-controlling-caregiving behaviors [21]. The impact of caregiving on OXTR methylation patterns in the child and the mother dyad was also investigated [22]. The quality of maternal engagement was found to be related to changes in child methylation between the 5- and 18-month visits. By contrast, maternal OXTR methylation remained stable in these periods, suggesting that infancy may represent a sensitive developmental period in which the oxytocin system is dynamic and responsive to the caregiving environment.

The child's age is also a factor known to influence the child's epigenetic profile in response to stress, and consequently, it may also impact the degree of the resemblance between mothers and children. This consideration arises from recognizing sensitive periods for life adversity's epigenetic impact [23]. Recent research has shown that the early age of exposure to life adversity significantly explained epigenetic variability, whereas this was not the case for the accumulation or the recency of adverse events [24]. The effect of timing has also been studied for the gene NR3C1 exon 1_F_, where early onset maltreated children showed significant hypermethylation when compared to non-maltreated children [25]. However, no significant differences in the methylation levels were observed between late-onset and non-maltreated children in the same genomic locations. Child age also indicates the time mother and child have shared the home space, implying that co-residence time might hold an epigenetic imprint for both mother and child. A recent study using the Horvath clock across the lifespan indicated that epigenetic aging correlations are dependent on familial co-residence, especially for parent–offspring pairs [26].

In this study, our first aim was to identify mother and child methylation similarity for the whole epigenome, given the scant evidence on this type of analysis. We also explored the similarity in the most common and relevant genes in empathy and stress literature: NR3C1, FKPB5, OXTR, SCL6A4, and BDNF [1, 23]. FKBP5 and NR3C1 regulate the hypothalamic–pituitary–adrenal (HPA) axis, and epigenetic changes have been frequently assessed in these genes [27]. The oxytocin receptor gene OXTR has been implicated in a range of primary social behaviors related to empathic bonding and attachment relationships [28]. OXTR also regulates responses to early life stress [29], complex social behaviors, and related psychopathologies characterized by socio-cognitive deficits [30, 31]. The serotonin transporter, encoded by the SLC6A4 gene, is responsible for serotonin reuptake into the presynaptic neuron related to the anti-depressant response [32]. Finally, the brain-derived neurotrophic factor BDNF regulates the development of the nervous system and the formation of appropriate synaptic connections involved in the HPA axis activity and stress regulation [33].

**Fig. 1:**
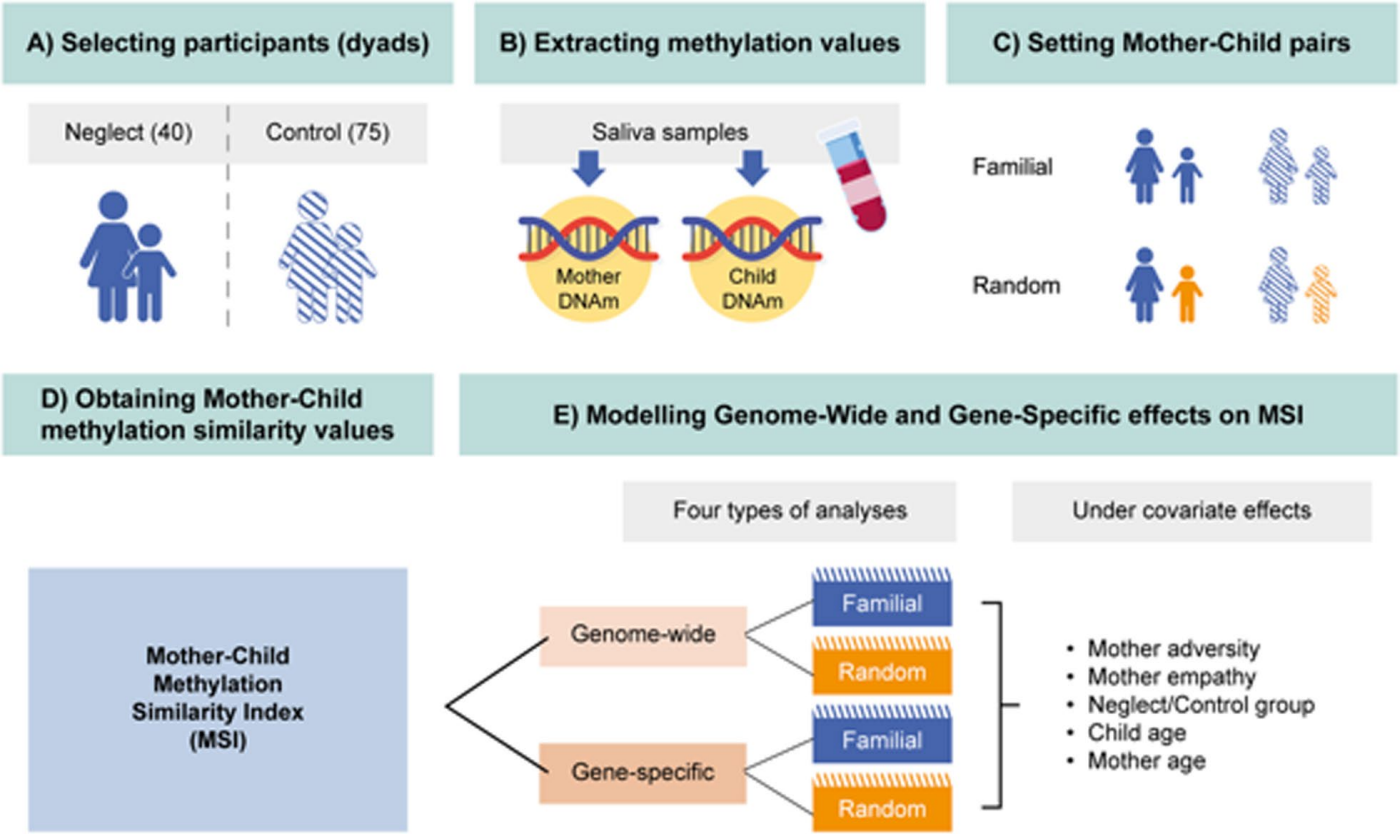
Design and analyses of the study. **A** Participants were selected according to their neglect/control parenting behavior. **B** Methylation values were obtained from mothers’ and children’s saliva samples. **C** Pairs were made by combinations of the mother–own child (familial pair) or mother–random child (random pair) in neglect and control dyads. **D** A mother–child *Methylation Similarity Index (MSI)* was obtained by correlating mother and child methylation values for each pair across the CpGs of the corresponding region. **E** Linear regression analyses against covariates were implemented at the whole epigenome and at specific genes for familial and random pairs

Our main objective was to assess whether the methylation similarity would be higher in mother–child pairs by familial links than in mother–child pairs by random links. The 115/115 saliva samples to measure DNAm were obtained from a group of 40 mother–child neglect dyads and a group of 75 mother–child non-neglect control dyads (see details of the design in the Method section, Fig. 1). We hypothesize that sharing an immediate family context and heritage links would provide more grounds for finding higher epigenetic similarities between mother–child familial pairs compared to random pairs. A *Methylation similarity index* (herein MSI) was obtained by computing correlation coefficients between methylation profiles within dyads, respecting the sequence of methylation values across CpGs for each gene and subject. It is important to note, that the MSI encompasses both the shared environmental influences between mothers and children and their genetic links. Previous studies on mother–child covariance tested the correlation between methylation levels in mothers and their children across the population [17]. In the referenced article [17], the methylation value is typically obtained from single CpG or from methylation values averaged within a specific genomic region of interest, such as the promoter region of a gene. In contrast, our approach explores the correlation between mothers and children across each CpG within the region of interest for each mother–child dyad separately. This procedure remains insensitive to variations in the overall methylation levels between individuals, placing emphasis on the finer adjustment of methylation convergence between the mother and the child. The analysis was performed for the genome-wide and the stress and empathy-related genes abovementioned.

To gain a deeper insight into the similarity of mother–child methylation profiles, our second aim was to investigate to what extent psychological covariates like Mother adversity, Mother empathy, belonging to the Neglect-control group, Child age, and Mother age modulate the MSI. We pursued this objective by employing multiple linear regression to investigate how changes in the mother–child MSI may be influenced by the covariates of interest, both at the genome-wide level and in NR3C1, FKPB5, OXTR, SCL6A4, and BDNF genes.

## Methods

### Participants

The study involved 115 mother–child dyads recruited from Municipal Social Services and Primary Health Centers: 40 in the neglect group and 75 in the non-neglect control group. Informed consent was obtained from the mothers and children, following the Code of Ethics of the World Medical Association. The inclusion criteria for both groups required to be biological mothers of children aged four months to eleven years without a history of foster care, premature birth, or medical complications. For the neglect group, verified cases of only substantiated physical neglect registered by Child Protective Services in the previous 12 months were included. For the control group, mothers with negative scores in the Maltreatment Classification System for severe neglect [34], and absence of Child Protection Services or Preventive Services records. As shown in Table 1, mothers in the neglect group were younger and with more children than mothers in the control group. The mean age of the target child and sex distribution were similar in both groups. Neglect mothers were less likely to live in two-parent families, had lower education levels, and received more financial assistance than mothers in the control group. Both groups had a moderate-high percentage of unemployment.

**Table 1:** Sociodemographic profile in the neglect and control groups

Variables	Neglect group *n* = 40 mothers	Control group *n* = 75 mothers	Comparison
	M (SD) or %	M (SD) or %	*t*(113)/*X*^2^
Mother current age	31.95 (6.66)	34.96 (6.18)	−2.13*
Age at child’s birth	27.89 (6.75)	31.07 (5.80)	−2.55*
Number of children	2.53 (1.34)	1.63 (0.61)	4.02***
Two-parent family %	48	77	6.31**
Level of education %			12.9**
Primary	77	43	
Secondary	20	53	
> Secondary	3	4	
Unemployment %	70	57	1.28
Financial assistance %	63	20	18.94***

Children	*n* = 40	*n* = 75	*t*(113)/*X*^2^
Age of target child (in years)	4.06 (2.77)	3.89 (2.15)	0.34
Female %	55	40	1.80

*p < .05; **p < .01; ***p < .001. Note: Group comparisons with mean scores were performed with t statistic, while those with percentage values were performed with Chi-square (*χ*^2^) statistic

### Psychological measures

*The Life Stress Scale LSS* [35]*,* was used to assess the mothers’ adverse life events experienced by the mother, making an adaptation of adverse childhood experiences to our risk population. It comprises 16 self-reported adverse events (e.g., divorce, economic pressure, chronic illness, eviction, unwanted pregnancy). Each item was rated on a categorical scale (no/yes occurrence). Its emotional impact on the participant was scored on a 3-point Likert scale (0 = no occurrence; 1 = little impact; 3 = very high impact). A cumulative score of mother adversity was obtained according to the emotional intensity of adverse events.

*The Empathic Concern (EC) scale* was extracted from the Interpersonal Reactivity Index (IRI) [36, 37]. It consisted of seven items extracted out of the 28 total items of the IRI, which measures an affective empathy characterized by feelings of warmth and concern for others (*α* = 0.60), which has shown a significant group difference in the neglect population [38]. Each aspect was scored on a 5-point Likert scale ranging from 1 = “Does not describe me well” to 5 = “Describe me very well”. The total score was calculated by adding the score for the corresponding seven items.

### Procedure

Social workers reported on the participants’ family characteristics and asked mothers for permission to be contacted by phone. Those mothers who gave permission were informed about the study and the ethics protocols, including the written acceptance. The use of the term “neglect” was avoided in these contacts. Mothers’ responses to the questionnaires and saliva samples were collected for each mother–child pair at home. Monetary compensation was given to the mothers after the session.

### Biosampling and DNA methylation analysis

The saliva was collected using the Real Saliva DNA Sample Collection Kit (Ref. RBMSAL01) for mothers and the Pediatric Genotek DNA Sample Collection Kit OC-175 for children. The DNA was extracted using the Maxwell extraction kit (Maxwell^®^ 16 Buccal Swab LEV DNA Purification Kit-Cat.#AS1295, Promega Corporation, Madison, WI, USA) at The University Hospital N.S. de Candelaria (Tenerife, Spain). The quality of DNA samples was assessed with the TapeStation instrument and their concentration and purity with spectrophotometry. Library preparation and methylation sequencing were conducted at the University of Michigan Epigenomics Core in Ann Arbor, United States. We performed an EWAS using the Illumina Human Methylation EPIC BeadChip. Given that DNA derived from saliva shows cellular heterogeneity, the value of the epithelial cells was calculated using the estimated LC function from ewastools R-package [39, 40], with the Houseman algorithm. The process (bisulfite conversion, hybridization, methylation value correction, probes and samples out of range removed) left us with 115 mother and child pairs and 771,785 probes of CpGs. Complete details of the procedure can be found in a previous study [13].

### Statistical analysis

As a previous step, M-transformed methylation values for the 771,785 CpGs for each mother and child were obtained. Then, for our first aim, the series of methylation values for each mother was correlated with the corresponding methylation values in her child across the whole epigenome, producing a mother–child Methylation similarity index (MSI) for each familial pair. Since the mother and child were measured in the same slide, this procedure controls for potential biases introduced by the experimental design (i.e., batch effects [41]). We also correlated methylation values across the whole epigenome in randomly paired mother and child for all possible random mother–child pairs in the same experimental slide. See Fig. 1 for a procedural representation of the design and analyses.

As shown in Fig. 1, an MSI was obtained for each familial and random pair. Before computing these correlations, the methylation values were corrected for the effect of leukocyte concentration, using the regression residuals as input for the subsequent analyses. The means of the MSI for the familial and random pairs in the whole epigenome were compared using two sample *t*-tests. These analyses were also performed for each target gene (NR3C1, FKBP5, OXTR, SLC6A4, and BDNF). Two-sample *t*-tests were also used to test differences in specific genes (five *t*-tests in total).

For our second aim, a multiple linear regression was performed to determine how Mother adversity, Mother empathy, the Neglect-control group, and the Child age, as independent variables could modulate the abovementioned mother–child MSI. Notice that while the conventional approach is to consider Neglect as the response variable to be characterized, here we consider it as another independent variable in the linear model to explain the MSI as a function of biological and environmental variables. Mother age in years was also included as a nuisance covariate in the linear regression to check its potential influence on the mother–child MSI. The dependent variable was each pair's MSI (between −1 and 1), normalized with the Fisher transform [42]. We fitted the model: MSI ~ Mother-adversity + Mother-empathy + Group (levels: neglect vs control) + Child-age + Mother-age + Intercept. The significant coefficients resulting from the linear regression indicate the effect of each covariate on the correlation scores. These analyses were performed both in familial and random pairs to control for the confounding effect and experimental noise that generates spurious correlations unrelated to the mother–child relationship. This regression analysis was implemented at the level of the whole epigenome and for the five genes of interest. Multiple comparison corrections were implemented by controlling with FDR correction (*q* = 0.05) [43]. For consistency, linear regressions were also implemented for random pairs to control for the possibility that these covariance effects could also be found.

## Results

### The mother–child similarity of methylation profiles in familial and random pairs

#### Whole epigenome analysis

**Fig. 2:**
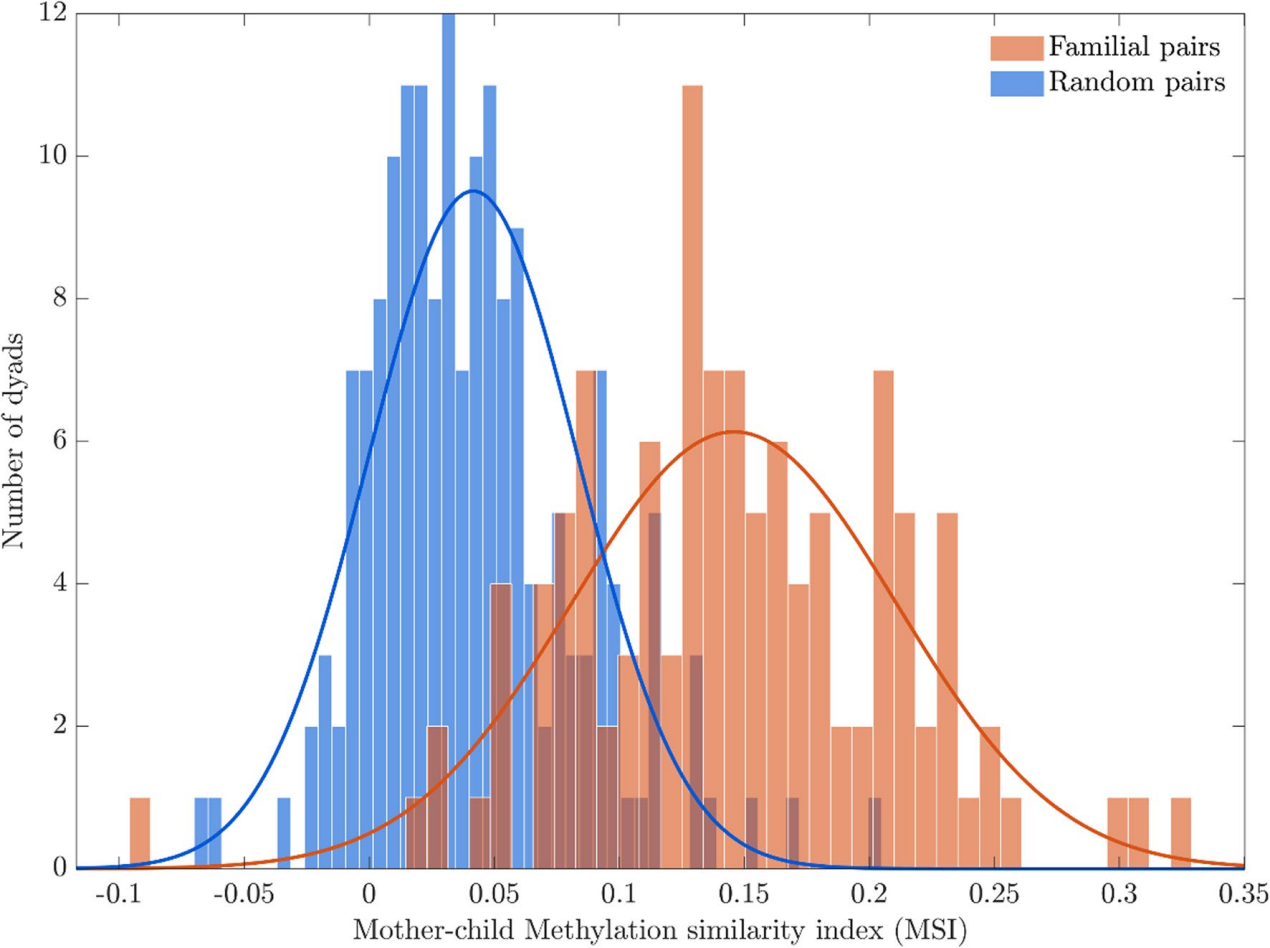
Histograms of the Methylation similarity index (MSI) for familial pairs (orange) and random pairs (blue). The continuous lines correspond to the histogram fits with a gaussian probability density

The means of MSI in the familial and random pairs significantly differed when compared with an independent sample *t*-test (*t*-statistics = 8.93 *p*-val < 0.001). As expected, familial pairs showed a higher MSI mean (correlation mean across dyads of 0.15) than random pairs (correlation mean across dyads of 0.05) (see Fig. 2).

#### Gene-specific analyses

Mean differences in the MSI between familial and random pairs were also obtained for the specific genes NR3C1, FKBP5, OXTR and BDNF but not for the SLC6A4 gene. The number of CpGs for each comparison, the mean of the MSI for familial and random pairs, and the result of the two sample *t*-tests are shown in Table 2.

**Table 2:** Descriptive information for each specific gene, including the number of CpGs, the mean Methylation similarity index (MSI) for familial and random mother–child pairs, and the *t*-statistics used to compare MSI in both groups

	Number of CpGs	Familial pairs	Random pairs	*t*-stat.
		Mean across dyads of the MSI		
Genes				
NR3C1	77	0.14	0.09	2.01*
FKBP5	46	0.22	0.09	3.56***
OXTR	18	0.08	0.02	1.70*
SLC6A4	31	0.08	0.07	0.66
BDNF	83	0.11	0.06	2.30**

*p < 0.05; **p < 0.01; ***p < 0.001

### Influence of psychological covariates on the mother–child methylation similarity profiles

#### Whole epigenome analysis

**Fig. 3:**
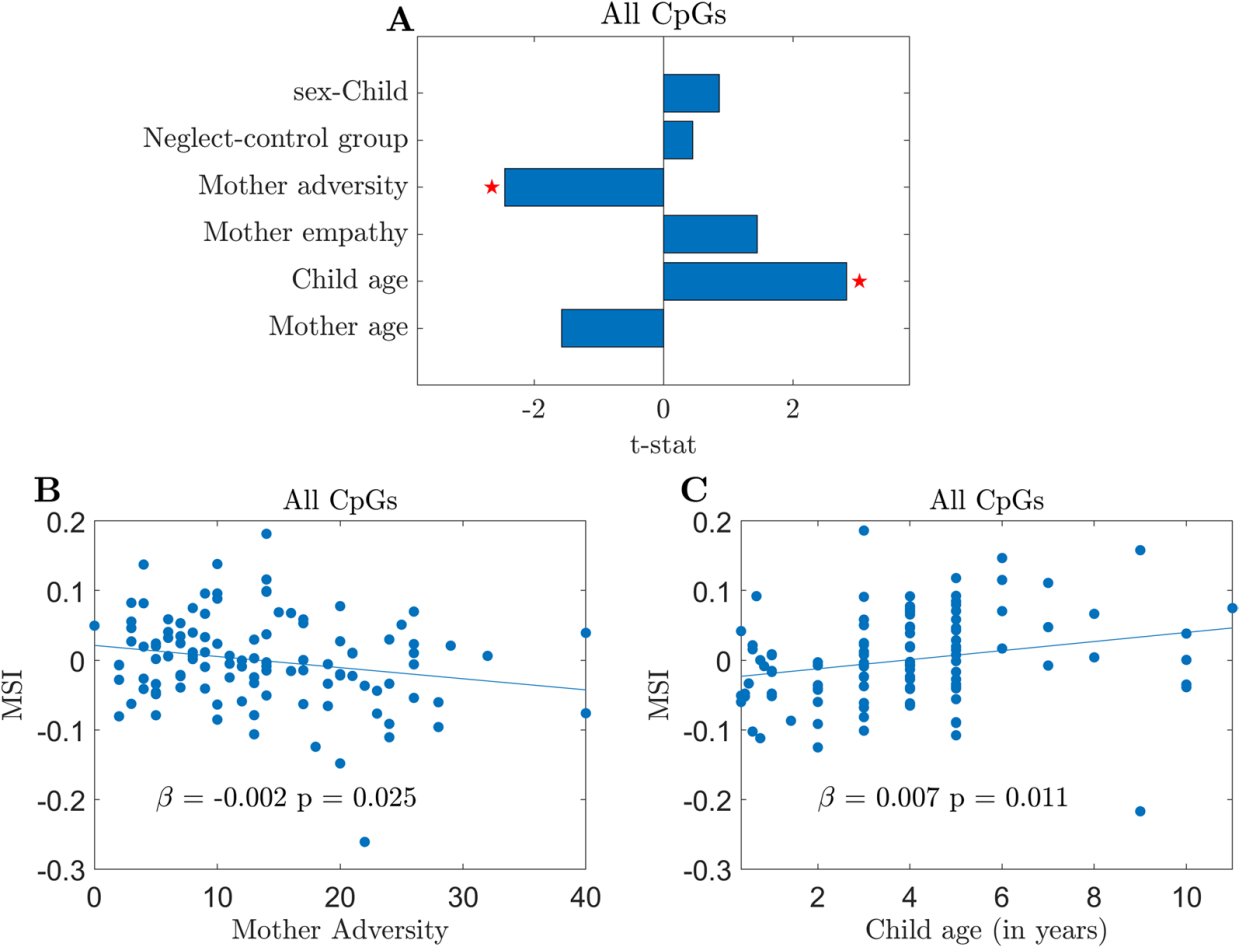
Linear regression shows the influence of the covariates of interest on the Methylation Similarity Index (MSI) in the genome-wide analyses. **A** Higher levels of Mother adversity were significantly associated with a lower MSI, whereas older Child age was significantly associated with a higher MSI. The length of the bar indicates the *t*-statistic value corresponding to each regression coefficient. The red asterisks denote statistical significance after multiple comparison corrections. **B** The scatter plot illustrates the effects of the Mother adversity on MSI. **C** The scatter plot shows the influence of the Child age on MSI. Every dot in the plot corresponds to a mother–child dyad when MSI was computed with all CpGs. Note that for each of the scatter plots, the contribution of the other significant variables in the model was removed from the MSI and the resulting residuals were used for visualization purpose

The linear regression model of the MSI against the covariates of interest showed *R*^2^ = 0.14 and an adjusted *R*^2^ = 0.10. The explained variance was significantly higher than the explained variance of the constant model *F*(106) = 3.55, *p* = 0.006. *t*-tests were implemented to evaluate the significance of each model’s coefficient. Significant effects (corrected for multiple comparisons) were found for the Mother adversity (*t* = −2.43, *p* = 0.017) and the Child age (*t* = 2.74, *p* = 0.007), as shown in Fig. 3A. The negative sign of the regression coefficient indicates that higher levels of Mother adversity were associated with a lower mother–child MSI. On the contrary, the positive sign indicates that the older Child age was associated with a higher MSI. The scatter plot displaying the effects for the significant covariates is presented in Figs. 3B and C. Note that for each of the scatter plots, the contribution of the other significant variable was removed from the MSI, and the resulting residuals were used for visualization.

The same linear regression model was applied for the random pairs to evaluate whether the exclusive familial link in the mother–child pairs drove the observed findings. Supporting the familial link hypothesis, the model showed non-significant results in random pairs with the explained variance of *R*^2^ = 0.03 and the adjusted explained variance of *R*^2^ = 0.01.

#### Gene-specific analysis

The method employed to assess the influence of covariates on the MSI for the entire epigenome was consistently applied for each candidate gene. From the five linear models, only two significantly explained the data variability, the one corresponding to OXTR (*F*(106) = 3.94, *p* = 0.002) and the corresponding to BDNF (*F*(106) = 3.47, *p* = 0.006). No significant linear relationships between the covariates and the mother–child MSI were found for the remaining genes. In addition, the linear models did not yield significant results when applied at the level of random pairs for all the studied genes.

**Fig. 4:**
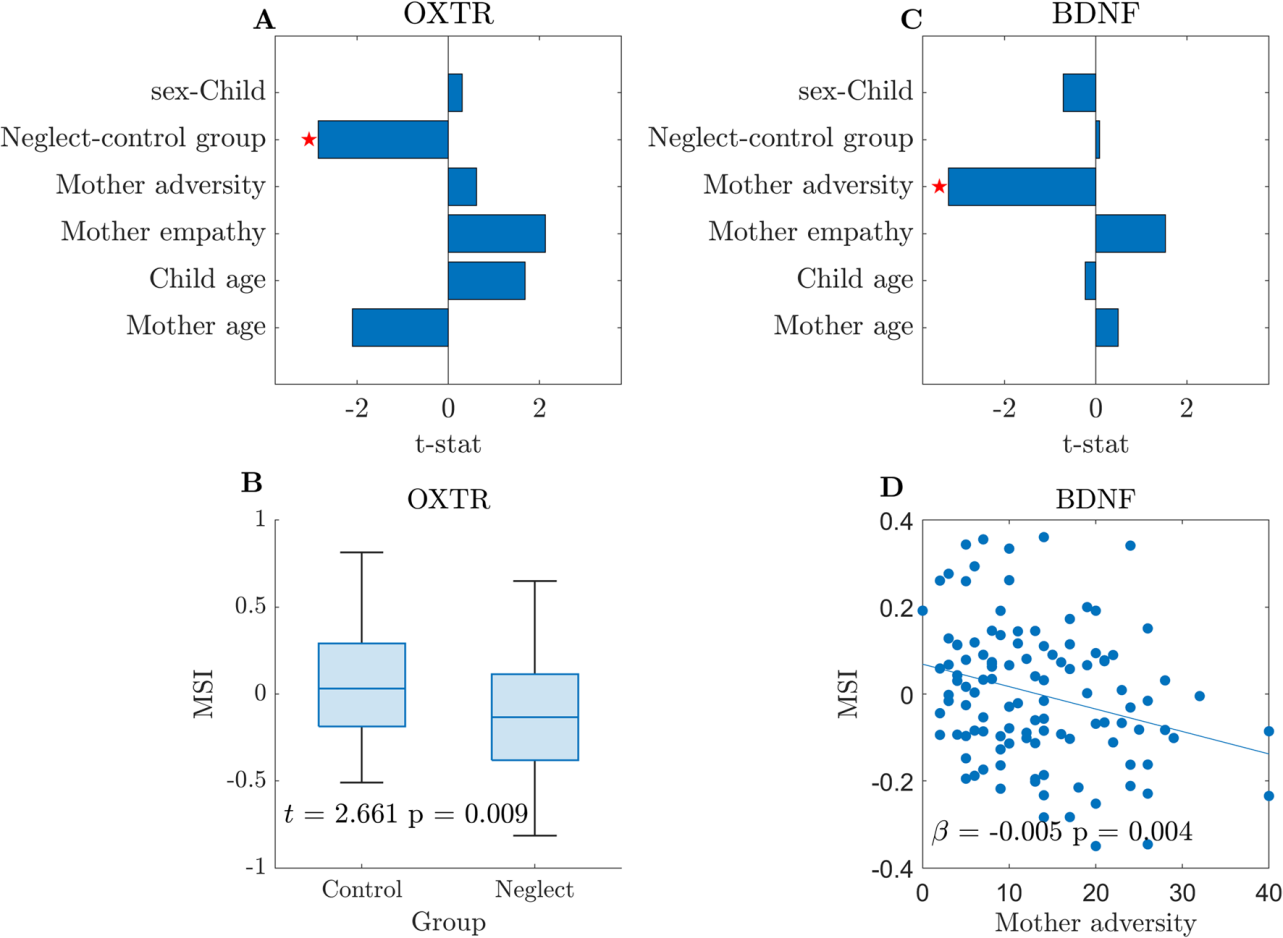
Linear model shows the influence of the covariates of interest on the Methylation similarity index for OXTR and BDNF. **A** The bar plot of the *t*-statistic for each regression coefficient shows that the Neglect group, as compared to the Control group was associated with a lower MSI in the OXTR gene. The red asterisks denote statistical significance after multiple comparison corrections. Of note, increases in Mother empathy positively correlated with the mother–child MSI in gene OXTR for an alpha level of 0.05. However, it did not survive multiple comparison corrections. **B** Bar plot across the levels of the factor group of the MSI in the OXTR gene. **C** The bar plot of the *t*-statistic for each regression coefficient, shows that the higher level of Mother adversity was associated with a lower MSI for the BDNF gene. **D** Scatter plot of Mother adversity versus MSI in BDNF gene. Every dot in the plot corresponds to a mother–child dyad

For those genes that resulted significant (OXTR and BDNF) in the previous analyses, *t*-tests were implemented to evaluate the significance of each model’s coefficient and the corresponding *p*-values were corrected for multiple comparisons (Fig. 4). For the OXTR gene, significant effects were found for the factor group (*t* = 2.66, *p* = 0.009), indicating that neglect dyads showed lower MSI than control dyads (see Fig. 4A). Of note, Mother empathy positively correlated with the mother–child MSI in the OXTR gene, for an alpha level of 0.05, however this effect did not survive multiple comparison corrections. The bar plot depicts the significant (Neglect-control group) comparison effects on the MSI in the OXTR gene (see Fig. 4B). For the BDNF gene, significant effects were found for Mother adversity (*t* = −2.98, *p* = 0.004), indicating that higher levels of adversity were associated with a lower MSI (see Fig. 4C). The scatter plot depicts the significant Mother adversity effects on the MSI (see Fig. 4D). Finally, an additional assessment of the influence of the child’s sex as a covariate on the MSI was conducted, showing no significant effect, both at the level of the entire epigenome and for individual genes.

## Discussion

This study examines dysfunctional mothering characterized by child physical neglect to explore convergence within the methylation profiles of both mothers and their children, covering the entire methylation array and specific genes. We also investigated how psychological covariables modulate the convergence in the mother–child methylation. In contrast to the standard approach employed, where covariation between two individuals is established through averaged methylation values in a gene or a set of CpGs, as observed in [17], our study adopted a novel strategy. We correlated the methylation profiles of mother and child while preserving the sequence of methylation values across CpGs for each individual rather than the average. This approach draws inspiration from methodologies used to compare methylation profiles between distinct human tissues within the same individual, such as peripheral blood and brain tissues [44–46].

We first examined whether there was a higher MSI in mother–child familial pairs than in random pairs. Consistent with our hypothesis, we found that in the entire epigenome, the mean of the similarity indexes was significantly higher in familial pairs with a genetic and environmental connection, than in randomly paired mothers and children. Furthermore, this finding was consistently supported when analyzing stress-regulation genes such as NR3C1, FKBP5, OXTR, and BDNF, although not in the case of the serotonin transporter SLC6A4 gen.

For our second objective, we examined the convergence of methylation profiles between mothers and children, both at the epigenome level and within specific genes. This convergence was significant only in the familial pairs and linked to maternal exposure to adverse events, child age as indicator of the time of co-residence, and neglect and control groups. The combined use of these covariates may shed more light on whether the observed mother–child transmission patterns are linked to the inheritance of epigenetic stress-related events or their shared environment [47]. Concerning the mothers’ DNAm effects from their exposure to adverse events, our results point out opposite intergenerational effects on their children's DNAm. At the epigenome level, we found that the higher the mother’s life stress, the lower the MSI between mothers and their children. This epigenome-wide opposite mother–child trend is also evident in two specific genes, BDNF and OXTR, where a reduced MSI was also found in presence of the covariate mother’s exposure to adverse life events. Another study also found an inversed pattern in methylation in the same site in a functional intronic region of stress-related gene FKBP5 in parents and offspring associated with preconceived maternal stress [15]. Regarding the OXTR receptor, our study revealed that neglected children had a lower MSI with their mothers compared to those children in control dyads. Typically, mothers who engage in neglectful caregiving often have experienced more life adversities in their own life [48]. Therefore, it is likely that they showed less epigenetic convergence with their children. A similar pattern was observed in one study with the oxytocin receptor gene [49]. This study found that mean OXTR methylation in mothers and newborns was positively associated with dyads in which mothers did not have experienced childhood maltreatment, but not in dyads in which mothers did.

A potential explanation of our results is that exposure to growing adversity increases methylation variability with a higher impact on the mother than on the child, probably due to her longer time of stress exposure. In that sense, our mothers may have more methylation changes than normative mothers increasing the epigenetic distance with their children under the effect of stressful life events. On a more theoretical grounds, the directional distinction observed between methylation profiles in the mothers and their children may be indicative of an adaptive response by offspring during the child's sensitive developmental period, aimed at mitigating the biological impacts of maternal exposure to trauma [15, 49]. Nevertheless, given that preconception and postnatal influences are both possible, further research involving mother–child dyads is necessary to delineate the conditions under which methylation patterns exhibit either negative or positive correlations with maternal adversity across generations. This will allow for a more comprehensive evaluation of our inverse trend.

A pattern of increasing mother–child MSI was obtained for the length of time that the child has lived with the mother (co-residence time), signaling the relevance of the mother–child shared environment. Interestingly, an early effect of epigenetic similarity was found from maternal-umbilical cord blood in certain promotor regions and in highly repeated elements, such as long interspersed nucleotide elements (LINE-1) and Alu, which may serve as surrogate markers for global DNA methylation [47]. Epigenetic coincidence has also been found in the context of post-traumatic stress in maternal blood, placental tissue, and umbilical cord blood in gen BDNF [50]. Building upon this initial similarity, our study has evidenced that continued mother–child residence facilitates a greater degree of methylation similarity, by exposing both members of the dyad to similar environmental factors. This epigenetic similarity becomes more pronounced as the duration of time shared together increases, as indicated by the child's age.

The co-residential effect on the within-dyad epigenetic similarity has also been demonstrated in different samples of monozygotic twins. In one study, the sample of twins was divided into two age groups, below and above 18 years old, differentiating twins living together from those living apart [51]. They found less epigenetic similarity in the group of older twins living apart, attributed consequently to the environmental differences or stochastic factors since the genetic component was automatically controlled by design. Other studies also found that correlations in methylation levels increased with the time twins lived together [26, 52]. Notably, no differences were found between monozygotic and dizygotic twins, supporting the relevance of the shared environment for greater epigenetic convergence rather than the genetic factor of being monozygotic. It was also found that epigenetic similarity decays (equally for monozygotic and dizygotic) with the time the twins lived apart. Although we did not control for another group of dyads living separately or considered genetic factors as a separate variable, our results go in the same direction since time of living together is a factor affecting the epigenetic convergence between mother and child.

Finally, a brief note is deserved to the empathy effect, in the direction that greater Mother empathy increased the mother–child MSI for the oxytocin receptor gene OXTR. Although this trend did not survive multiple comparison corrections, it theoretically underscores the relevance of empathic care since OXTR has been implicated in a range of early social behaviors related to bonding and attachment relationships [28, 29]. Maternal empathic care tentatively appears to be involved in the OXTR regulation of developmental experiences displayed during early mother–child interactions. These positive exchanges have been shown to play a critical role in establishing secure child attachment and ensuring subsequent health and well-being [53, 54].

Despite being the only epigenetic similarity study with well-characterized mother–child dyads in the neglect condition and evaluated in tandem, our study has limitations. Difficulty in finding neglectful mother–child dyads limited our sample size to 115 mother–child dyads, which is relatively small in epigenetic studies but is larger than the average size of the dyadic studies cited (mean = 102 dyads) [15–17, 47, 55]. The significant results achieved through epigenome-wide analysis and rigorous methodological controls also bolster our confidence that the sample size has not increased in Type 2 errors. Nevertheless, future studies must replicate our findings with larger sample sizes, as well as evaluate the effect of genetic variants (Single Nucleotide Polymorphism) affecting the differentially methylated CpGs to provide additional explanatory data on the biological transmission. Additional longitudinal studies might also help to understand the dynamic nature of methylation changes over time in mother–child dyads associated with co-residence. Using our innovative methodology, which focused on the mother–child methylation profile similarity as the dependent variable rather than individual methylation averages, may offer a more comprehensive way of mapping mother–child epigenetic coincidences.

In conclusion, the analysis of the dyadic epigenetic similarity in the methylation profiles has allowed us to confirm the greater methylation correlation in familial pairs than in random pairs both epigenome-wide and in stress-relevant genes NR3C1, FKBP5, OXTR and BDNF. Moreover, using different covariates as modulating factors in the intergenerational methylation process offers a valuable opportunity to gain insights into development-dependent adaptations that are influenced by both hereditary and environmental factors, significantly observed only in biological dyads. Two opposing driving forces have been identified influencing the epigenome array and genes BDNF and OXTR: one leading to reduced epigenetic convergence, and the other supporting a greater convergence. Mother adversity and belonging to the neglect group lead to reduced epigenetic convergence revealing a lower potential of such risk factors for mother-to-child methylation transmission. By contrast, mother–child time of co-residence, indexed by child age, promotes increased epigenetic convergence, evidencing a higher potential for methylation transmission during the ontogeny. The same convergence tendency, although not reached a significant level, showed the mother’s empathic concern trait in the OXTR gene.

Our findings have twofold implications for child well-being. On the positive side, children of mothers exposed to life adversity or neglect did not necessarily inherit a direct replica of their methylation patterns. The other is concerning due to the influence of time spent living together, which affects similarity with the mother and potentially increases the risk of inheriting an epigenetic profile associated with future dysfunctional parenting patterns. This underscores the importance of the 'the earlier, the better' recommendation by the Child Protection System, which is not always followed. In response to this alarm, it is urgent to prevent the progressive epigenetic impact of chronic and unnoticed situations of child neglect resulting from longer shared exposure to an insensitive and unstimulating immediate environment. To this aim, it is important to expand the possibilities for an early diagnosis of the neglect condition in both the mother, who has experienced trauma, and her newborn child, being performed at primary care screenings. Next, for those cases with early signs of neglect risk, training in mother–child stimulation and empathic care should be incorporated into targeted interventions to break the cycle of intergenerational transmission of child neglect and prevent subsequent negative outcomes.

## Data Availability

Data are available from the authors upon request to the authors.
